# The Practice of Experimental Psychology: An Inevitably Postmodern Endeavor

**DOI:** 10.3389/fpsyg.2020.612805

**Published:** 2021-01-12

**Authors:** Roland Mayrhofer, Christof Kuhbandner, Corinna Lindner

**Affiliations:** Department of Psychology, University of Regensburg, Regensburg, Germany

**Keywords:** postmodernism, experimental psychology, experiment, methodology, philosophy of science

## Abstract

The aim of psychology is to understand the human mind and behavior. In contemporary psychology, the method of choice to accomplish this incredibly complex endeavor is the experiment. This dominance has shaped the whole discipline from the self-concept as an empirical science and its very epistemological and theoretical foundations, via research practice and the scientific discourse to teaching. Experimental psychology is grounded in the scientific method and positivism, and these principles, which are characteristic for modern thinking, are still upheld. Despite this apparently stalwart adherence to modern principles, experimental psychology exhibits a number of aspects which can best be described as facets of postmodern thinking although they are hardly acknowledged as such. Many psychologists take pride in being “real natural scientists” because they conduct experiments, but it is particularly difficult for psychologists to evade certain elements of postmodern thinking in view of the specific nature of their subject matter. Postmodernism as a philosophy emerged in the 20th century as a response to the perceived inadequacy of the modern approach and as a means to understand the complexities, ambiguities, and contradictions of the times. Therefore, postmodernism offers both valuable insights into the very nature of experimental psychology and fruitful ideas on improving experimental practice to better reflect the complexities and ambiguities of human mind and behavior. Analyzing experimental psychology along postmodern lines begins by discussing the implications of transferring the scientific method from fields with rather narrowly defined phenomena—the natural sciences—to a much broader and more heterogeneous class of complex phenomena, namely the human mind and behavior. This ostensibly modern experimental approach is, however, *per se* riddled with postmodern elements: (re-)creating phenomena in an experimental setting, including the hermeneutic processes of generating hypotheses and interpreting results, is no carbon copy of “reality” but rather an active construction which reflects irrevocably the pre-existing ideas of the investigator. These aspects, analyzed by using postmodern concepts like hyperreality and simulacra, did not seep in gradually but have been present since the very inception of experimental psychology, and they are necessarily inherent in its philosophy of science. We illustrate this theoretical analysis with the help of two examples, namely experiments on free will and visual working memory. The postmodern perspective reveals some pitfalls in the practice of experimental psychology. Furthermore, we suggest that accepting the inherently fuzzy nature of theoretical constructs in psychology and thinking more along postmodern lines would actually clarify many theoretical problems in experimental psychology.

## Introduction

Postmodernism is, in essence, an attempt to achieve greater clarity in our perception, thinking, and behavior by scrutinizing their larger contexts and preconditions, based on the inextricably intertwined levels of both the individual and the society. Psychology also studies the human mind and behavior, which indicates that psychology should dovetail with postmodern approaches. In the 1990s and early 2000s, several attempts were made to introduce postmodern thought as potentially very fruitful ideas into general academic psychology (Jager, 1991; Kvale, 1992; Holzman and Morss, 2000; Holzman, 2006). However, overall they were met with little response.

Postmodern thoughts have been taken up by several fringe areas of academic psychology, e.g., psychoanalysis (Leffert, 2007; Jiménez, 2015; but see Holt, 2005), some forms of therapy and counseling (Ramey and Grubb, 2009; Hansen, 2015), humanistic (Krippner, 2001), feminist and gender (Hare-Mustin and Marecek, 1988; Sinacore and Enns, 2005), or cultural psychology (Gemignani and Peña, 2007).

However, there is resistance against suggestions to incorporate postmodern ideas into the methodology and the self-perception of psychology as academic—and scientific!—discipline. In fact, postmodern approaches are often rejected vehemently, sometimes even very vocally. For instance, Gergen (2001) argued that the “core tenets” of postmodernism are not at odds with those of scientific psychology but rather that they can enrich the discipline by opening up new possibilities. His suggestions were met with reservation and were even outright rejected on the following grounds: postmodernism, “like anthrax of the intellect, if *allowed* [our italics] into mainstream psychology, […] will poison the field” (Locke, 2002, 458), that it “wishes to return psychology to a prescientific subset of philosophy” (Kruger, 2002, 456), and that psychology “needs fewer theoretical and philosophical orientations, not more” (Hofmann, 2002, 462; see also Gergen’s, 2001, replies to the less biased and more informed commentaries on his article).

In the following years, and continuing the so-called science wars of the 1990s (Segerstråle, 2000), several other attacks were launched against a perceived rise or even dominance of postmodern thought in psychology. Held(2007; see also the rebuttal by Martin and Sugarman, 2009) argued that anything postmodern would undermine rationality and destroy academic psychology. Similarly, postmodernism was identified—together with “radical environmentalism” and “pseudoscience” among other things—as a “key threat to scientific psychology” (Lilienfeld, 2010, 282), or as “inimical to progress in the psychology of science” (Capaldi and Proctor, 2013, 331). The following advice was given to psychologists: “We [psychologists] should also push back against the pernicious creep of these untested concepts into our field” (Tarescavage, 2020, 4). Furthermore, the term “postmodern” is even employed as an all-purpose invective in a popular scientific book by psychologist Steven Pinker (2018).

Therefore, it seems that science and experimental psychology on the one hand and postmodern thinking on the other are irreconcilable opposites. However, following Gergen (2001) and Holtz (2020), we argue that this dichotomy is only superficial because postmodernism is often misunderstood. A closer look reveals that experimental psychology contains many postmodern elements. Even more, there is reason to assume that a postmodern perspective may be beneficial for academic psychology: First, the practice of experimental psychology would be improved by integrating postmodern thinking because it reveals a side of the human psyche for which experimental psychology is mostly blind. Second, the postmodern perspective can tell us much about the epistemological and social background of experimental psychology and how this affects our understanding of the human psyche.

## A Postmodern Perspective on Experimental Psychology

### Experimental Psychology and the Modern Scientific Worldview

It lies within the nature of humans to try to find out more about themselves and their world, but the so-called Scientific Revolution of the early modern period marks the beginning of a new era in this search for knowledge. The Scientific Revolution, which has led to impressive achievements in the natural sciences and the explanation of the physical world (e.g., Olby et al., 1991; Henry, 1997; Cohen, 2015; Osterlind, 2019), is based on the following principle: to “measure what can be measured and make measurable what cannot be measured.” This famous appeal—falsely attributed to Galileo Galilei but actually from the 19th century (Kleinert, 2009)—illustrates the two fundamental principles of modern science: First, the concept of “measurement” encompasses the idea that phenomena can be quantified, i.e., expressed numerically. Second, the concept of “causal connections” pertains to the idea that consistent, non-random relationships can be established between measurable phenomena. Quantification allows that relationships between phenomena can be expressed, calculated, and predicted in precise mathematical and numerical terms.

However, there are two important issues to be aware of. First, while it is not difficult to measure “evident” aspects, such as mass and distance, more complex phenomena cannot be measured easily. In such cases, it is therefore necessary to find ways of making these “elusive” phenomena measurable. This can often only be achieved by reducing complex phenomena to their simpler—and measurable!—elements. For instance, in order to measure memory ability precisely, possible effects of individual preexisting knowledge which introduce random variance and thus impreciseness have to be eliminated. Indeed, due to this reason, in many memory experiments, meaningless syllables are used as study material.

Second, it is not difficult to scientifically prove a causal relationship between a factor and an outcome if the relationship is simple, that is, if there is only one single factor directly influencing the outcome. In such a case, showing that a manipulation of the factor causes a change in the outcome is clear evidence for a causal relationship because there are no other factors which may influence the outcome as well. However, in situations where many factors influence an outcome in a complex, interactive way, proving a causal relationship is much more difficult. To prove the causal effect of one factor in such a situation the effects of all other factors—called confounding factors from the perspective of the factor of interest—have to be eliminated so that a change in the outcome can be truly attributed to a causal effect of the factor of interest. However, this has an important implication: The investigator has to divide the factors present in a given situation into interesting versus non-interesting factors with respect to the current context of the experiment. Consequently, while experiments reveal something about local causal relationships, they do not necessarily provide hints about the net effect of all causal factors present in the given situation.

The adoption of the principles of modern science has also changed psychology. Although the beginnings of psychology—as the study of the *psyche*—date back to antiquity, psychology as an academic discipline was established in the mid to late 19th century. This enterprise was also inspired by the success of the natural sciences, and psychology was explicitly modeled after this example by Wilhelm Wundt—the “father of experimental psychology”—although he emphasized the close ties to the humanities as well. The experiment quickly became the method of choice. There were other, more hermeneutic approaches during this formative phase of modern psychology, such as psychoanalysis or introspection according to the Würzburg School, but their impact on academic psychology was limited. Behaviorism emerged as a direct reaction against these perceived unscientific approaches, and its proponents emphasized the scientific character of their “new philosophy of psychology.” It is crucial to note that in doing so they also emphasized the importance of the experiment and the necessity of quantifying directly observable behavior in psychological research. Behaviorism quickly became a very influential paradigm which shaped academic psychology. Gestalt psychologists, whose worldview is radically different from behaviorism, also relied on experiments in their research. Cognitive psychology, which followed, complemented, and partly superseded behaviorism, relies heavily on the experiment as a means to gain insight into mental processes, although other methods such as modeling are employed as well. Interestingly, there is a fundamental difference between psychoanalysis and humanistic psychology, which do not rely on the experiment, and the other above-mentioned approaches as the former focus on the psychic functioning of individuals, whereas the latter focus more on global laws of psychic functioning across individuals. This is reflected in the fact that psychological laws in experimental psychology are established on the arithmetic means across examined participants—a difference we will elaborate on later in more detail. Today, psychology is the *scientific*—in the sense of empirical-quantitative—study of the human mind and behavior, and the experiment is often considered the gold standard in psychological research (e.g., Mandler, 2007; Goodwin, 2015; Leahey, 2017).

The experiment is closely associated with the so-called scientific method (Haig, 2014; Nola and Sankey, 2014) and the epistemological tenets philosophy of positivism—in the sense as Martin (2003); Michell (2003), and Teo (2018) explain—which sometimes exhibit characteristics of naïve empiricism. Roughly speaking, the former consists of observing, formulating hypotheses, and testing these hypotheses in experiments. The latter postulates that knowledge is based on sensory experience, that it is testable, independent of the investigator and therefore objective as it accurately depicts the world as it is. This means that in principle all of reality can not only be measured but eventually be entirely explained by science. This worldview is attacked by postmodern thinkers who contend that the world is far more complex and that the modern scientific approach cannot explain all of reality and its phenomena.

### The Postmodern Worldview

Postmodern thinking (e.g., Bertens, 1995; Sim, 2011; Aylesworth, 2015) has gained momentum since the 1980s, and although neither the term “postmodernism” nor associated approaches can be defined in a unanimous or precise way, they are characterized by several intertwined concepts, attitudes, and aims. The most basic trait is a general skepticism and the willingness to question literally everything from the ground up—even going so far as to question not only the foundation of any idea, but also the question itself. This includes the own context, the chosen premises, thinking, and the use of language. Postmodernism therefore has a lot in common with science’s curiosity to understand the world: the skeptical attitude paired with the desire to discover how things really are.

Postmodern investigations often start by looking at the language and the broader context of certain phenomena due to the fact that language is the medium in which many of our mental activities—which subsequently influence our behavior—take place. Thus, the way we talk reveals something about how and why we think and act. Additionally, we communicate about phenomena using language, which in turn means that this discourse influences the way we think about or see those phenomena. Moreover, this discourse is embedded in a larger social and historical context, which also reflects back on the use of language and therefore on our perception and interpretation of certain phenomena.

Generally speaking, postmodern investigations aim at detecting and explaining how the individual is affected by societal influences and their underlying, often hidden ideas, structures, or mechanisms. As these influences are often fuzzy, contradictory, and dependent on their context, the individual is subject to a multitude of different causalities, and this already complex interplay is further complicated by the personal history, motivations, aims, or ways of thinking of the individual. Postmodernism attempts to understand all of this complexity as it is in its entirety.

The postmodern approaches have revealed three major general tendencies which characterize the contemporary world: First, societies and the human experience since the 20th century have displayed less coherence and conversely a greater diversity than the centuries before in virtually all areas, e.g., worldviews, modes of thinking, societal structures, or individual behavior. Second, this observation leads postmodern thinkers to the conclusion that the grand narratives which dominated the preceding centuries and shaped whole societies by providing frames of references have lost—at least partially—their supremacy and validity. Examples are religious dogmas, nationalism, industrialization, the notion of linear progress—and modern science because it works according to certain fundamental principles. Third, the fact that different but equally valid perspectives, especially on social phenomena or even whole worldviews, are possible and can coexist obviously affects the concepts of “truth,” “reality,” and “reason” in such a way that these concepts lose their immutable, absolute, and universal or global character, simply because they are expressions and reflections of a certain era, society, or worldview.

At this point, however, it is necessary to clarify a common misconception: Interpreting truth, reality, or reason as relative, subjective, and context-dependent—as opposed to absolute, objective, and context-independent—does naturally neither mean that anything can be arbitrarily labeled as true, real, or reasonable, nor, vice versa, that something cannot be true, real, or reasonable. For example, the often-quoted assumption that postmodernism apparently even denies the existence of gravity or its effects as everything can be interpreted arbitrarily or states that we cannot elucidate these phenomena with adequate accuracy because everything is open to any interpretation (Sokal, 1996), completely misses the point.

First, postmodernism is usually not concerned with the laws of physics and the inanimate world as such but rather focuses on the world of human experience. However, the phenomenon itself, e.g., gravity, is not the same as our scientific knowledge of phenomena—our chosen areas of research, methodological paradigms, data, theories, and explanations—or our perception of phenomena, which are both the results of human activities. Therefore, the social context influences our scientific knowledge, and in that sense scientific knowledge is a social construction (Hodge, 1999).

Second, phenomena from human experience, although probably more dependent on the social context than physical phenomena, cannot be interpreted arbitrarily either. The individual context—such as the personal history, motivations, aims, or worldviews—determines whether a certain behavior makes sense for a certain individual in a certain situation. As there are almost unlimited possible backgrounds, this might seem completely random or arbitrary from an overall perspective. But from the perspective of an individual the phenomenon in question may be explained entirely by a theory for a specific—and not universal—context.

As described above, the postmodern meta-perspective directly deals with human experience and is therefore especially relevant for psychology. Moreover, any discipline—including the knowledge it generates—will certainly benefit from understanding its own (social) mechanisms and implications. We will show below that postmodern thinking not only elucidates the broader context of psychology as an academic discipline but rather that experimental psychology exhibits a number of aspects which can best be described as facets of postmodern thinking although they are not acknowledged as such.

### The Postmodern Context of Experimental Psychology

Paradoxically, postmodern elements have been present since the very beginning of experimental psychology although postmodernism gained momentum only decades later. One of the characteristics of postmodernism is the transplantation of certain elements from their original context to new contexts, e.g., the popularity of “Eastern” philosophies and practices in contemporary “Western” societies. These different elements are often juxtaposed and combined to create something new, e.g., new “westernized” forms of yoga (Shearer, 2020).

Similarly, the founders of modern academic psychology took up the scientific method, which was originally developed in the context of the natural sciences, and transplanted it to the study of the human *psyche* in the hope to repeat the success of the natural sciences. By contrast, methods developed specifically in the context of psychology such as psychoanalysis (Wax, 1995) or introspection according to the Würzburg School (Hackert and Weger, 2018) have gained much less ground in academic psychology. The way we understand both the *psyche* and psychology has been shaped to a great extent by the transfer of the principles of modern science, namely quantitative measurement and experimental methods, although it is not evident *per se* that this is the best approach to elucidate mental and behavioral phenomena. Applying the methods of the natural sciences to a new and different context, namely to phenomena pertaining to the human *psyche*, is a truly postmodern endeavor because it juxtaposes two quite distinct areas and merges them into something new—experimental psychology.

The postmodern character of experimental psychology becomes evident on two levels: First, the subject matter—the human *psyche*—exhibits a postmodern character since mental and behavioral phenomena are highly dependent on the idiosyncratic contexts of the involved individuals, which makes it impossible to establish unambiguous general laws to describe them. Second, experimental psychology itself displays substantial postmodern traits because both its method and the knowledge it produces—although seemingly objective and rooted in the modern scientific worldview—inevitably contain postmodern elements, as will be shown below.

### The Experiment as Simulacrum

The term “simulacrum” basically means “copy,” often in the sense of “inferior copy” or “phantasm/illusion.” However, in postmodern usage “simulacrum” has acquired a more nuanced and concrete meaning. “Simulacrum” is a key term in the work of postmodern philosopher Jean Baudrillard, who arguably presented the most elaborate theory on simulacra (1981/1994). According to Baudrillard, a simulacrum “is the reflection of a profound [‘real’] reality” (16/6). Simulacra, however, are more than identical carbon copies because they gain a life of their own and become “real” in the sense of becoming an own entity. For example, the personality a pop star shows on stage is not “real” in the sense that it is their “normal,” off-stage personality, but it is certainly “real” in the sense that it is perceived by the audience even if they are aware that it might be an “artificial” personality. Two identical cars can also be “different” for one might be used as a means of transportation while the other might be a status symbol. Even an honest video documentation of a certain event is not simply a copy of the events that took place because it lies within the medium video that only certain sections can be recorded from a certain perspective. Additionally, the playback happens in other contexts as the original event, which may also alter the perception of the viewer.

The post-structuralist—an approach closely associated with postmodernism—philosopher Roland Barthes pointed out another important aspect of simulacra. He contended that in order to understand something—an “object” in Barthes’ terminology—we necessarily create simulacra because we “*reconstruct* [our italics] an ‘object’ in such a way as to manifest thereby the rules of functioning [⋯] of this object” (Barthes, 1963, 213/214). In other words, when we investigate an object—any phenomenon, either material, mental, or social—we have to perceive it first. This means that we must have some kind of mental representation of the phenomenon/object—and it is crucial to note that this representation is not the same thing as the “real” object itself. All our mental operations are therefore not performed on the “real” object but on mental representations of the object. We decompose a phenomenon in order to understand it, that is, we try to identify its components. In doing so, we effect a change in the object because our phenomenon is no longer the original phenomenon “as it is” for we are performing a mental operation on it, thereby transforming the original phenomenon. Identifying components may be simple, e.g., dividing a tree into roots, trunk, branches, and leaves may seem obvious or even “natural” but it is nevertheless us as investigators who create this structure—the tree itself is probably not aware of it. Now that we have established this structure, we are able to say that the tree consists of several components and name these components. Thus, we have introduced “new” elements *into our understanding* of the tree. This is the important point, even though the elements, i.e., the branches and leaves themselves “as they are,” have naturally always been “present.” Our understanding of “tree” has therefore changed completely because a tree is now something which is composed of several elements. In that sense, we have changed the original phenomenon by adding something—and this has all happened in our thinking and not in the tree itself. It is also possible to find different structures and different components for the tree, e.g., the brown and the green, which shows that we construct this knowledge.

Next, we can investigate the components to see how they interact with and relate to each other and to the whole system. Also, we can work out their functions and determine the conditions under which a certain event will occur. We can even expand the scope of our investigation and examine the tree in the context of its ecosystem. But no matter what we do or how sophisticated our investigation becomes, everything said above remains true here, too, because neither all these actions listed above nor the knowledge we gain from them are the object itself. Rather, we have added something to the object and the more we know about our object, the more knowledge we have constructed. This addition is what science—gaining knowledge—is all about. Or in the words of Roland Barthes: “the simulacrum is intellect added to object, and this addition has an anthropological value, in that it is man himself, his history, his situation, his freedom and the very resistance which nature offers to his mind” (1963/1972, 214/215).

In principle, this holds truth regarding all scientific investigations. But the more complex phenomena are, the more effort and personal contribution is required on behalf of the investigator to come up with structures, theories, or explanations. Paraphrasing Barthes: When dealing with complex phenomena, more intellect must be added to the object, which means in turn that there are more possibilities for different approaches and perspectives, that is, the constructive element becomes larger. As discussed previously, this does not mean that investigative and interpretative processes are arbitrary. But it is clear from this train of thought that “objectivity” or “truth” in a “positivist,” naïve empiricist “realist,” or absolute sense are not attainable. Nevertheless, we argue here that this is not a drawback, as many critics of postmodernism contend (see above), but rather an advantage because it allows more accurate scientific investigations of true-to-life phenomena, which are typically complex in the case of psychology.

The concepts of simulacra by Baudrillard and Barthes can be combined to provide a description of the experiment in psychology. Accordingly, our understanding of the concept of the “simulacrum” entails that scientific processes—indeed all investigative processes—necessarily need to duplicate the object of their investigation in order to understand it. In doing so, constructive elements are necessarily introduced. These elements are of a varying nature, which means that investigations of one and the same phenomenon may differ from each other and different investigations may find out different things about the phenomenon in question. These investigations then become entities on their own—in the Baudrillardian sense—and therefore simulacra.

In a groundbreaking article on “the meaning and limits of exact science” physicist Max Planck stated that “[a]n experiment is a question which science poses to nature, and a measurement is the recording of nature’s answer” (Planck, 1949, 325). The act of “asking a question” implies that the person asking the question has at least a general idea of what the answer might look like (Heidegger, 1953, §2). For example: When asking someone for their name, we obviously do not know what they are called, but we assume that they have a name and we also have an idea of how the concept “name” works. Otherwise we could not even conceive, let alone formulate, and pose our question. This highlights how a certain degree of knowledge and understanding of a concept is necessary so that we are able to ask questions about it. Likewise, we need to have a principal idea or assumption of possible mechanisms if we want to find out how more complex phenomena function. It is—at least at the beginning—irrelevant whether these ideas are factually correct or entirely wrong, for without them we would be unable to approach our subject matter in the first place.

The context of the investigator—their general worldview, their previous knowledge and understanding, and their social situation—obviously plays an important part in the process of forming a question which can be asked in the current research context. Although this context may be analyzed along postmodern lines in order to find out how it affects research, production of knowledge, and—when the knowledge is applied—possible (social) consequences, there is a much more profound implication pertaining to the very nature of the experiment as a means to gain knowledge.

Irrespective of whether it is a simple experiment in physics such as Galileo Galilei’s or an experiment on a complex phenomenon from social or cognitive psychology, the experiment is a situation which is specifically *designed* to answer a certain type of questions, usually causal relationships, such as: “Does A causally affect B?” Excluding the extremely complex discussion on the nature of causality and causation (e.g., Armstrong, 1997; Pearl, 2009; Paul and Hall, 2013), it is crucial to note that we need the experiment as a tool to answer this question. Although we may theorize about a phenomenon and infer causal relationships simply by observing, we cannot—at least according to the prevailing understanding of causality in the sciences—prove causal relationships without the experiment.

The basic idea of the experiment is to create conditions which differ in only one single factor which is suspected as a causal factor for an effect. The influence of all other potential causal relationships is kept identical because they are considered as confounding factors which are irrelevant from the perspective of the research question of the current experiment. Then, if a difference is found in the outcome between the experimental conditions, this is considered as proof that the aspect in question exerts indeed a causal effect. This procedure and the logic behind it are not difficult to understand. However, a closer look reveals that this is actually far from simple or obvious.

To begin with, an experiment is nothing which occurs “naturally” but a situation created for a specific purpose, i.e., an “artificial” situation, because other causal factors exerting influence in “real” life outside the laboratory are deliberately excluded and considered as “confounding” factors. This in itself shows that the experiment contains a substantial postmodern element because instead of creating something it rather *re-*creates it. This re-creation is of course based on phenomena from the “profound” reality—in the Baudrillardian sense—since the explicit aim is to find out something about this profound reality and not to create something new or something else. However, as stated above, this re-creation must contain constructive elements reflecting the presuppositions, conceptual-theoretical assumptions, and aims of the investigator. By focusing on one factor and by reducing the complexity of the profound reality, the practical operationalization and realization thus reflect both the underlying conceptual structure and the anticipated outcome as they are specifically designed to test for the suspected but hidden or obscured causal relationships.

At this point, another element becomes relevant, namely the all-important role of language, which is emphasized in postmodern thinking (e.g., Harris, 2005). Without going into the intricacies of semiotics, there is an explanatory gap (Chalmers, 2005)—to borrow a phrase from philosophy of mind—between the phenomenon on the one hand and the linguistic and/or mental representation of it on the other. This relationship is far from clear and it is therefore problematic to assume that our linguistic or mental representations—our words and the concepts they designate—are identical with the phenomena themselves. Although we cannot, at least according to our present knowledge and understanding, fully bridge this gap, it is essential to be aware of it in order to avoid some pitfalls, as will be shown in the examples below.

Even a seemingly simple word like “tree”—to take up once more our previous example—refers to a tangible phenomenon because there are trees “out there.” However, they come in all shapes and sizes, there are different kinds of trees, and every single one of them may be labeled as “tree.” Furthermore, trees are composed of different parts, and the leaf—although part of the tree—has its own word, i.e., linguistic and mental representation. Although the leaf is part of the tree—at least according to *our* concepts—it is unclear whether “tree” also somehow encompasses “leaf.” The same holds true for the molecular, atomic, or even subatomic levels, where there “is” no tree. Excluding the extremely complex ontological implications of this problem, it has become clear that we are referring to a certain level of granularity when using the word “tree.” The level of granularity reflects the context, aims, and concepts of the investigator, e.g., an investigation of the rain forest as an ecosystem will ignore the subatomic level.

How does this concern experimental psychology? Psychology studies intangible phenomena, namely mental and behavioral processes, such as cognition, memory, learning, motivation, emotion, perception, consciousness, etc. It is important to note that these terms designate *theoretical* constructs as, for example, memory cannot be observed directly. We may provide the subjects of an experiment a set of words to learn and observe later how many words they reproduce correctly. A theoretical construct therefore describes such relationships between stimulus and behavior, and we may *draw conclusions* from this observable data *about* memory. But neither the observable behavior of the subject, the resulting data, nor our conclusions are identical with memory itself.

This train of thought demonstrates the postmodern character of experimental psychology because we construct our knowledge. But there is more to it than that: Even by trying to define a theoretical construct as exactly as possible—e.g., memory as “the process of maintaining information over time” (Matlin, 2012, 505) or “the means by which we retain and draw on our past experiences to use this information in the present” (Sternberg and Sternberg, 2011, 187)—the explanatory gap between representation and phenomenon cannot be bridged. Rather, it becomes even more complicated because theoretical constructs are composed of other theoretical constructs, which results in some kind of self-referential circularity where constructs are defined by other constructs which refer to further constructs. In the definitions above, for instance, hardly any key term is self-evident and unambiguous for there are different interpretations of the constructs “process,” “maintaining,” “information,” “means,” “retain,” “draw on,” “experiences,” and “use” according to their respective contexts. Only the temporal expressions “over time,” “past,” and “present” are probably less ambiguous here because they are employed as non-technical, everyday terms. However, the definitions above are certainly not entirely incomprehensible—in fact, they are rather easy to understand in everyday language—and it is *quite clear* what the *authors intend* to *express*. The italics indicate constructive elements, which demonstrates that attempts to give a precise definition in the language of science result in fuzziness and self-reference.

Based on a story by Jorge Luis Borges, Baudrillard (1981) found an illustrative allegory: a map so precise that it portrays everything in perfect detail—but therefore inevitably so large that it shrouds the entire territory it depicts. Similarly, Taleb (2007) coined the term “ludic fallacy” for mistaking the model/map—in our context: experiments in psychology—for the reality/territory, that is, a mental or behavioral phenomenon. Similar to the functionality of a seemingly “imprecise” map which contains only the relevant landmarks so the user may find their way, the fuzziness of language poses no problems in everyday communication. So why is it a problem in experimental psychology? Since the nature of theoretical constructs in psychology lies precisely in their very fuzziness, the aim of reaching a high degree of granularity and precision in experimental psychology seems to be unattainable (see the various failed attempts to create “perfect” languages which might depict literally everything “perfectly,” e.g., Carapezza and D’Agostino, 2010).

Without speculating about ontic or epistemic implications, it is necessary to be aware of the explanatory gap and to refrain from identifying the experiment and the underlying operationalization with the theoretical construct. Otherwise, this gap is “filled” unintentionally and uncontrollably if the results of an experiment are taken as valid proof for a certain theoretical construct, which is actually fuzzy and potentially operationalizable in a variety of ways. If this is not acknowledged, words, such as “memory,” become merely symbols devoid of concrete meaning, much like a glass bead game—or in postmodern terminology: a hyperreality.

### Experiments and Hyperreality

“Hyperreality” is another key term in the work of Jean Baudrillard (1981) and it denotes a concept closely related to the simulacrum. Accordingly, in modern society the simulacra are ubiquitous and they form a system of interconnected simulacra which refer to each other rather than to the real, thereby possibly hiding or replacing the real. Consequently, the simulacra become real in their own right and form a “more real” reality, namely the hyperreality. One may or may not accept Baudrillard’s conception, especially the all-embracing social and societal implications, but the core concept of “hyperreality” is nevertheless a fruitful tool to analyze experimental psychology. We have already seen that the experiment displays many characteristics of a simulacrum, so it is not surprising that the concept of hyperreality is applicable here as well, although in a slightly different interpretation than Baudrillard’s.

The hyperreal character of the experiment can be discussed on two levels: the experiment itself and the discourse wherein it is embedded.

On the level of the experiment itself, two curious observations must be taken into account. First, and in contrast to the natural sciences where the investigator is human and the subject matter (mostly) non-human and usually inanimate, in psychology both the investigator and the subject matter are human. This means that the subjects of the experiment, being autonomous persons, are not malleable or completely controllable by the investigator because they bring their own background, history, worldview, expectations, and motivations. They interpret the situation—the experiment—and act accordingly, but not necessarily in the way the investigator had planned or anticipated (Smedslund, 2016). Therefore, the subjects create their own versions of the experiment, or, in postmodern terminology, a variety of simulacra, which may be more or less compatible with the framework of the investigator. This holds true for all subjects of an experiment, which means that the experiment as a whole may also be interpreted as an aggregation of interconnected simulacra—a hyperreality.

The hyperreal character becomes even more evident because what contributes in the end to the interpretation of the results of the experiment are not the actual performances and results of the individual subjects as they were intended by them but rather how their performances and results are handled, seen, and interpreted by the investigator. Even if the investigator tries to be as faithful as possible and aims at an exact and unbiased measurement—i.e., an exact copy—there are inevitably constructive elements which introduce uncertainty into the experiment. Investigators can never be certain what the subjects were actually doing and thinking so they must necessarily work with interpretations. Or in postmodern terms: Because the actual performances and results of the subjects are not directly available the investigators must deal with simulacra. These simulacra become the investigators’ reality and thus any further treatment—statistical analyses, interpretations, or discussions—becomes a hyperreality, that is, a set of interconnected simulacra which have become “real.”

On the level of the discourse wherein the experiment is embedded, another curious aspect also demonstrates the hyperreal character of experimental psychology. Psychology is, according to the standard definition, the scientific study of mental and behavioral processes of the individual (e.g., Gerrig, 2012). This definition contains two actually contradictory elements. On the one hand, the focus is on processes of the individual. On the other hand, the—scientific—method to elucidate these processes does not look at individuals *per se* but aggregates their individual experiences and transforms them into a “standard” experience. The results from experiments, our knowledge of the human psyche, reflect psychological functioning at the level of the mean across individuals. And even if we assume that the mean is only an estimator and not an exact description or prediction, the question remains open how de-individualized observations are related to the experience of an individual. A general mechanism, a law—which was discovered by abstracting from a multitude of individual experiences—is then (*re*-)imposed in the opposite direction back onto the individual. In other words, a simulacrum—namely, the result of an experiment—is viewed and treated as reality, thus becoming hyperreal. Additionally, and simply because it is considered universally true, this postulated law acquires thereby a certain validity and “truth”—often irrespective of its actual, factual, or “profound” truth—on its own. Therefore, it can become impossible to distinguish between “profound” and “simulacral” truth, which is the hallmark of hyperreality.

### Measuring the Capacity of the Visual Working Memory

Vision is an important sensory modality and there is extensive research on this area (Hutmacher, 2019). Much of our daily experience is shaped by seeing a rich and complex world around us, and it is therefore an interesting question how much visual information we can store and process. Based on the development of a seminal experimental paradigm, Luck and Vogel (1997) have shown that visual working memory has a storage capacity of about four items. This finding is reported in many textbooks (e.g., Baddeley, 2007; Parkin, 2013; Goldstein, 2015) and has almost become a truism in cognitive psychology.

The experimental paradigm developed by Luck and Vogel (1997) is a prime example of an experiment which closely adheres to the scientific principles outlined above. In order to make a very broad and fuzzy phenomenon measurable, simple abstract forms are employed as visual stimuli—such as colored squares, triangles, or lines, usually on a “neutral,” e.g., gray, background—which can be counted in order to measure the capacity of visual working memory. Reducing the exuberant diversity of the “outside visual world” to a few abstract geometric forms is an extremely artificial situation. The obvious contrast between simple geometrical forms and the rich panorama of the “real” visual world illustrates the pitfalls of controlling supposed confounding variables, namely the incontrollable variety of the “real” world and how we see it. Precisely by abstracting and by excluding potential confounding variables it is possible to count the items and to make the capacity of the visual working memory measurable. But in doing so the original phenomenon—seeing the whole world—is lost. In other words: A simulacrum has been created.

The establishment of the experimental paradigm by Luck and Vogel has led to much research and sparked an extensive discussion how the limitation to only four items might be explained (see the summaries by Brady et al., 2011; Luck and Vogel, 2013; Ma et al., 2014; Schurgin, 2018). However, critically, several studies have shown that the situation is different when real-world objects are used as visual stimuli rather than simple abstract forms, revealing that the capacity of the visual working memory is higher for real-world objects (Endress and Potter, 2014; Brady et al., 2016; Schurgin et al., 2018; Robinson et al., 2020; also Schurgin and Brady, 2019). Such findings show that the discourse about the mechanisms behind the limitations of the visual working memory is mostly about an artificial phenomenon which has no counterpart in “reality”—the perfect example of a hyperreality.

This hyperreal character does not mean that the findings of Luck and Vogel (1997) or similar experiments employing artificial stimuli are irrelevant or not “true.” The results are true—but it is a *local* truth, only valid for the specific context of *specific* experiments, and not a *global* truth which applies to the visual working memory *in general*. That is, speaking about “visual working memory” based on the paradigm of Luck and Vogel is a mistake because it is actually about “visual working memory for simple abstract geometrical forms in front of a gray background.”

### Free Will and Experimental Psychology

The term “free will” expresses the idea of having “a significant kind of *control* [italics in the original] over one’s actions” (O’Connor and Franklin, 2018, n.p.). This concept has occupied a central position in Western philosophy since antiquity because it has far-reaching consequences for our self-conception as humans and our position in the world, including questions of morality, responsibility, and the nature of legal systems (e.g., Beebee, 2013; McKenna and Pereboom, 2016; O’Connor and Franklin, 2018). Being a topic of general interest, it is not surprising that experimental psychologists have tried to investigate free will as well.

The most famous study was conducted by Libet et al. (1983), and this experiment has quickly become a focal point in the extensive discourse on free will because it provides empirical data and a scientific investigation. Libet et al.’s experiment seems to show that the subjective impression when persons consciously decide to act is in fact preceded by objectively measurable but unconscious physical processes. This purportedly proves that our seemingly voluntary actions are actually predetermined by physical processes because the brain has unconsciously reached a decision already before the person becomes aware of it and that our conscious intentions are simply grafted onto it. Therefore, we do not have a free will, and consequently much of our social fabric is based on an illusion. Or so the story goes.

This description, although phrased somewhat pointedly, represents a typical line of thought in the discourse on free will (e.g., the prominent psychologists Gazzaniga, 2011; Wegner, 2017; see Kihlstrom, 2017, for further examples).

Libet’s experiment sparked an extensive and highly controversial discussion: For some authors, it is a refutation or at least threat to various concepts of free will, or, conversely, an indicator or even proof for some kind of material determinism. By contrast, other authors deny that the experiment refutes or counts against free will. Furthermore, a third group—whose position we adopt for our further argumentation—denies that Libet’s findings are even relevant for this question at all (for summaries of this complex and extensive discussion and various positions including further references see Nahmias, 2010; Radder and Meynen, 2013; Schlosser, 2014; Fischborn, 2016; Lavazza, 2016; Schurger, 2017). Libet’s own position, although not entirely consistent, opposes most notions of free will (Roskies, 2011; Seifert, 2011). Given this background, it is not surprising that there are also numerous further experimental studies on various aspects of this subject area (see the summaries by Saigle et al., 2018; Shepard, 2018; Brass et al., 2019).

However, we argue that this entire discourse is best understood along postmodern lines as hyperreality and that Libet’s experiment itself is a perfect example of a simulacrum. A closer look at the concrete procedure of the experiment shows that Libet actually asked his participants to move their hand or finger “at will” while their brain activity was monitored with an EEG. They were instructed to keep watch in an introspective manner for the moment when they felt the “urge” to move their hand and to record this moment by indicating the clock-position of a pointer. This is obviously a highly artificial situation where the broad and fuzzy concept of “free will” is abstracted and reduced to the movement of the finger, the only degree of freedom being the moment of the movement. The question whether this is an adequate operationalization of free will is of paramount importance, and there are many objections that Libet’s setup fails to measure free will at all (e.g., Mele, 2007; Roskies, 2011; Kihlstrom, 2017; Brass et al., 2019).

Before Libet, there was no indication that the decision when to move a finger might be relevant for the concept of free will and the associated discourse. The question whether we have control over our actions referred to completely different levels of granularity. Free will was discussed with respect to questions such as whether we are free to live our lives according to our wishes or whether we are responsible for our actions in social contexts (e.g., Beebee, 2013; McKenna and Pereboom, 2016; O’Connor and Franklin, 2018), and not whether we lift a finger now or two seconds later. Libet’s and others’ jumping from very specific situations to far-reaching conclusions about a very broad and fuzzy theoretical construct illustrates that an extremely wide chasm between two phenomena, namely moving the finger and free will, is bridged in one fell swoop.

In other words, Libet’s experiment is a simulacrum as it duplicates a phenomenon from our day-to-day experience—namely free will—but in doing so the operationalization alters and reduces the theoretical construct. The outcome is a questionable procedure whose relationship to the phenomenon is highly controversial. Furthermore, the fact that, despite its tenuous connection to free will, Libet’s experiment sparked an extensive discussion on this subject reveals the hyperreal nature of the entire discourse because what is being discussed is not the actual question—namely free will—but rather a simulacrum. Everything else—the arguments, counter-arguments, follow-up experiments, and their interpretations—built upon Libet’s experiment are basically commentaries to a simulacrum and not on the real phenomena. Therefore, a hyperreality is created where the discourse revolves around entirely artificial phenomena, but where the arguments in this discussion refer back to and affect the real as suggestions are made to alter the legal system and our ideas of responsibility—which, incidentally, is not a question of empirical science but of law, ethics, and philosophy.

All of the above is not meant to say that this whole discourse is meaningless or even gratuitous—on the contrary, our understanding of the subject matter has greatly increased. Although our knowledge of free will has hardly increased, we have gained much insight into the hermeneutics and methodology—and pitfalls!—of *investigations* of free will, possible consequences on the individual and societal level, and the workings of scientific discourses. And this is exactly what postmodernism is about.

## Discussion

As shown above, there are a number of postmodern elements in the practice of experimental psychology: The prominent role of language, the gap between the linguistic or mental representation and the phenomenon, the “addition of intellect to the object,” the simulacral character of the experiment itself in its attempt to re-create phenomena, which necessarily transforms the “real” phenomenon due to the requirements of the experiment, and finally the creation of a hyperreality if experiments are taken as the “real” phenomenon and the scientific discourse becomes an exchange of symbolic expressions referring to the simulacra created in experiments, replacing the real. All these aspects did not seep gradually into experimental psychology in the wake of postmodernism but have been present since the very inception of experimental psychology as they are necessarily inherent in its philosophy of science.

Given these inherent postmodern traits in experimental psychology, it is puzzling that there is so much resistance against a perceived “threat” of psychology’s scientificness. Although a detailed investigation of the reasons lies outside the scope of this analysis, we suspect there are two main causes: First, an insufficient knowledge of the history of science and understanding of philosophy of science may result in idealized concepts of a “pure” natural science. Second, lacking familiarity with basic tenets of postmodern approaches may lead to the assumption that postmodernism is just an idle game of arbitrary words. However, “science” and “postmodernism” and their respective epistemological concepts are not opposites (Gergen, 2001; Holtz, 2020). This is especially true for psychology, which necessarily contains a social dimension because not only the investigators are humans but also the very subject matter itself.

The (over-)reliance on quantitative-experimental methods in psychology, often paired with a superficial understanding of the philosophy of science behind it, has been criticized, either from the theoretical point of view (e.g., Bergmann and Spence, 1941; Hearnshaw, 1941; Petrie, 1971; Law, 2004; Smedslund, 2016) or because the experimental approach has failed to produce reliable, valid, and relevant applicable knowledge in educational psychology (Slavin, 2002). It is perhaps symptomatic that a textbook teaching the principles of science for psychologists does not contain even one example from experimental psychology but employs only examples from physics, plus Darwin’s theory of evolution (Wilton and Harley, 2017).

On the other hand, the postmodern perspective on experimental psychology provides insight into some pitfalls, as illustrated by the examples above. On the level of the experiment, the methodological requirements imply the creation of an artificial situation, which opens up a gap between the phenomenon as it is in reality and as it is concretely operationalized in the experimental situation. This is not a problem *per se* as long as is it clear—and clearly communicated!—that the results of the experiment are only valid in a certain context. The problems begin if the movement of a finger is mistaken for free will. Similarly, being aware that local causalities do not explain complex phenomena such as mental and behavioral processes in their entirety also prevents (over-) generalization, especially if communicated appropriately. These limitations make it clear that the experiment should not be made into an absolute or seen as the only valid way of understanding the *psyche* and the world.

On the level of psychology as an academic discipline, any investigation must select the appropriate level of granularity and strike a balance between the methodological requirements and the general meaning of the theoretical concept in question to find out something about the “real” world. If the level of granularity is so fine that results cannot be tied back to broader theoretical constructs rather than providing a helpful understanding of our psychological functioning, academic psychology is in danger of becoming a self-referential hyperreality.

The postmodern character of experimental psychology also allows for a different view on the so-called replication crisis in psychology. Authors contending that there is no replication crisis often employ arguments which exhibit postmodern elements, such as the emphasis on specific local conditions in experiments which may explain different outcomes of replication studies (Stroebe and Strack, 2014; Baumeister, 2019). In other words, they invoke the simulacral character of experiments. This explanation may be valid or not, but the replication crisis has shown the limits of a predominantly experimental approach in psychology.

Acknowledging the postmodern nature of experimental psychology and incorporating postmodern thinking explicitly into our research may offer a way out of this situation. Our subject matter—the *psyche*—is extremely complex, ambiguous, and often contradictory. And postmodern thinking has proven capable of successfully explaining such phenomena (e.g., Bertens, 1995; Sim, 2011; Aylesworth, 2015). Thus, paradoxically, by accepting and considering the inherently fuzzy nature of theoretical constructs, they often become much clearer (Ronzitti, 2011). Therefore, thinking more along postmodern lines in psychology would actually sharpen the theoretical and conceptual basis of experimental psychology—all the more as experimental psychology has inevitably been a postmodern endeavor since its very beginning.

## Author Contributions

RM, CK, and CL developed the idea for this article. RM drafted the manuscript. CK and CL provided feedback and suggestions. All authors approved the manuscript for submission.

## Conflict of Interest

The authors declare that the research was conducted in the absence of any commercial or financial relationships that could be construed as a potential conflict of interest.

## References

[B1] Armstrong D. M. (1997). *A World of States of Affairs.* Cambridge: CUP.

[B2] Aylesworth G. (2015). “Postmodernism,” in *The Stanford Encyclopedia of Philosophy*, ed. Zalta E. N. Available online at: https://plato.stanford.edu/entries/postmodernism/

[B3] Baddeley A. (2007). *Working Memory, Thought, and Action.* Oxford: OUP.

[B4] Barthes R. (1963). “L’activité structuraliste,” in *Essais Critiques* (pp. 215–218). Paris: Éditions du Seuil. [“Structuralist activity.” Translated by R. Howard (1972). In *Critical Essays*, ed. Barthes R. (Evanston: Northern University Press), 213–220].

[B5] Baudrillard J. (1981). *Simulacres et Simulation*. Paris: Galilée. [*Simulacra and Simulation*. Translated by S. F. Glaser (1994). Ann Arbor: The University of Michigan Press.]

[B6] Baumeister R. F. (2019). “Self-control, ego depletion, and social psychology’s replication crisis,” in *Surrounding Self-control* (Appendix to chap. 2), ed. Mele A. (New York, NY: OUP). 10.31234/osf.io/uf3cn

[B7] Beebee H. (2013). *Free Will: An Introduction.* New York, NY: Palgrave Macmillan.

[B8] Bergmann G. Spence K. W. (1941). Operationism and theory in psychology. *Psychol. Rev.* 48 1–14. 10.1037/h0054874

[B9] Bertens H. (1995). *The Idea of the Postmodern. A History.* London: Routledge.

[B10] Brady T. F. Konkle T. Alvarez G. A. (2011). A review of visual memory capacity: beyond individual items and toward structured representations. *J. Vis.* 11 1–34. 10.1167/11.5.4 PMC3405498 21617025

[B11] Brady T. F. Störmer V. S. Alvarez G. A. (2016). Working memory is not fixed-capacity: more active storage capacity for real-world objects than for simple stimuli. *Proc. Natl. Acad. Sci. U.S.A.* 113 7459–7464. 10.1073/pnas.1520027113 27325767 PMC4941470

[B12] Brass M. Furstenberg A. Mele A. R. (2019). Why neuroscience does not disprove free will. *Neurosci. Biobehav. Rev.* 102 251–263. 10.1016/j.neubiorev.2019.04.024 31059730

[B13] Capaldi E. J. Proctor R. W. (2013). “Postmodernism and the development of the psychology of science,” in *Handbook of the Psychology of Science*, eds Feist G. J. Gorman M. E. (New York, NY: Springer), 331–352.

[B14] Carapezza M. D’Agostino M. (2010). Logic and the myth of the perfect language. *Logic Philos. Sci.* 8 1–29. 10.1093/oso/9780190869816.003.0001

[B15] Chalmers D. (2005). “Phenomenal concepts and the explanatory gap,” in *Phenomenal Concepts and Phenomenal Knowledge. New Essays on Consciousness and Physicalism*, eds Alter T. Walter S. (Oxford: OUP), 167–194. 10.1093/acprof:oso/9780195171655.003.0009

[B16] Cohen H. F. (2015). *The Rise of Modern Science Explained: A Comparative History.* Cambridge: CUP.

[B17] Endress A. D. Potter M. C. (2014). Large capacity temporary visual memory. *J. Exp. Psychol. Gen.* 143 548–565. 10.1037/a0033934 23937181 PMC3974584

[B18] Fischborn M. (2016). Libet-style experiments, neuroscience, and libertarian free will. *Philos. Psychol.* 29 494–502. 10.1080/09515089.2016.1141399

[B19] Gazzaniga M. S. (2011). *Who’s in Charge? Free Will and the Science of the Brain.* New York, NY: Ecco.

[B20] Gemignani M. Peña E. (2007). Postmodern conceptualizations of culture in social constructionism and cultural studies. *J. Theor. Philos. Psychol.* 27–28 276–300. 10.1037/h0091297

[B21] Gergen K. J. (2001). Psychological science in a postmodern context. *Am. Psychol.* 56 803–813. 10.1037/0003-066X.56.10.803 11675987

[B22] Gergen K. J. (2002). Psychological science: to conserve or create? *Am. Psychol.* 57 463–464. 10.1037/0003-066X.57.6-7.463 12094461

[B23] Gerrig R. J. (2012). *Psychology and Life*, 20th Edn Boston: Pearson.

[B24] Goldstein E. B. (2015). *Cognitive Psychology: Connecting Mind, Research and Everyday Experience.* Stamford: Cengage Learning.

[B25] Goodwin C. J. (2015). *A History of Modern Psychology*, 5th Edn Hoboken, NJ: Wiley.

[B26] Hackert B. Weger U. (2018). Introspection and the Würzburg school: implications for experimental psychology today. *Eur. Psychol.* 23 217–232. 10.1027/1016-9040/a000329

[B27] Haig B. D. (2014). *Investigating the Psychological World: Scientific Method in the Behavioral Sciences.* Cambridge, MA: MIT Press.

[B28] Hansen J. T. (2015). The relevance of postmodernism to counselors and counseling practice. *J. Ment. Health Counsel.* 37 355–363. 10.17744/MEHC.37.4.06

[B29] Hare-Mustin R. T. Marecek J. (1988). The meaning of difference: gender theory, postmodernism, and psychology. *Am. Psychol.* 43 455–464. 10.1037//0003-066X.43.6.455

[B30] Harris R. (2005). *The Semantics of Science.* London: Continuum.

[B31] Hearnshaw L. S. (1941). Psychology and operationism. *Aust. J. Psychol. Philos.* 19 44–57. 10.1080/00048404108541506

[B32] Heidegger M. (1953). *Sein und Zeit* (7. Aufl.). Tübingen: Niemeyer. [*Being and Time*. Translated by J. Stambaugh, revised by D. J. Schmidt (2010). Albany: SUNY Press.]

[B33] Held B. S. (2007). *Psychology’s Interpretive Turn: The Search for Truth and Agency in Theoretical and Philosophical Psychology.* Washington, DC: APA.

[B34] Henry J. (1997). *The Scientific Revolution and the Origins of Modern Science.* Basingstoke: Macmillan.

[B35] Hodge B. (1999). The Sokal ‘Hoax’: some implications for science and postmodernism. *Continuum J. Media Cult. Stud.* 13 255–269. 10.1080/10304319909365797

[B36] Hofmann S. G. (2002). More science, not less. *Am. Psychol.* 57:462 10.1037//0003-066X.57.6-7.462a 12094459

[B37] Holt R. R. (2005). “The menace of postmodernism to a psychoanalytic psychology,” in *Relatedness, Self-definition and Mental Representation: Essays in Honor of Sidney J. Blatt*, eds Auerbach J. S. Levy K. N. Schaffer C. E. (London: Routledge), 288–302. 10.4324/9780203337318_chapter_18

[B38] Holtz P. (2020). Does postmodernism really entail a disregard for the truth? Similarities and differences in postmodern and critical rationalist conceptualizations of truth, progress, and empirical research methods. *Front. Psychol.* 11:545959 10.3389/fpsyg.2020.545959 PMC7527490 33041913

[B39] Holzman L. (2006). Activating postmodernism. *Theory Psychol.* 16 109–123. 10.1177/0959354306060110

[B40] Holzman L. Morss J. (eds). (2000). *Postmodern Psychologies, Societal Practice, and Political Life.* New York, NY: Routledge.

[B41] Hutmacher F. (2019). Why is there so much more research on vision than on any other sensory modality? *Front. Psychol.* 10:2246. 10.3389/fpsyg.2019.02246 31636589 PMC6787282

[B42] Jager B. (1991). Psychology in a postmodern era. *J. Phenomenol. Psychol.* 22 60–71. 10.1163/156916291X00046

[B43] Jiménez J. P. (2015). Psychoanalysis in postmodern times: some questions and challenges. *Psychoanal. Inquiry* 35 609–624. 10.1080/07351690.2015.1055221

[B44] Kihlstrom J. F. (2017). Time to lay the Libet experiment to rest: commentary on Papanicolaou (2017). *Psycho. Conscious. Theory Res. Pract.* 4 324–329. 10.1037/cns0000124

[B45] Kleinert A. (2009). Der messende Luchs. *NTM. Z. Gesch. Wiss. Tech. Med.* 17 199–206. 10.1007/s00048-009-0335-4

[B46] Krippner S. (2001). “Research methodology in humanistic psychology in the light of postmodernity,” in *The Handbook of Humanistic Psychology: Leading Edges in Theory, Research, and Practice*, eds Schneider K. J. Bugental J. F. Pierson J. F. (Thousand Oaks, CA: SAGE Publications), 290–304. 10.4135/9781412976268.n22

[B47] Kruger D. J. (2002). The deconstruction of constructivism. *Am. Psychol.* 57 456–457. 10.1037/0003-066X.57.6-7.456 12094453

[B48] Kvale S. (ed.) (1992). *Psychology and Postmodernism.* London: SAGE.

[B49] Lavazza A. (2016). Free will and neuroscience: from explaining freedom away to new ways of operationalizing and measuring it. *Front. Hum. Neurosci.* 10:262. 10.3389/fnhum.2016.00262 27313524 PMC4887467

[B50] Law J. (2004). *After Method: Mess in Social Science Research.* London: Routledge.

[B51] Leahey T. H. (2017). *A History of Psychology: From Antiquity to Modernity*, 8th Edn New York, NY: Routledge.

[B52] Leffert M. (2007). A contemporary integration of modern and postmodern trends in psychoanalysis. *J. Am. Psychoanal. Assoc.* 55 177–197. 10.1177/00030651070550011001 17432496

[B53] Libet B. Gleason C. A. Wright E. W. Pearl D. K. (1983). Time of conscious intention to act in relation to onset of cerebral activity (readiness-potential). The unconscious initiation of a freely voluntary act. *Brain* 106 623–642. 10.1093/brain/106.3.623 6640273

[B54] Lilienfeld S. O. (2010). Can psychology become a science? *Pers. Individ. Differ.* 49 281–288. 10.1016/j.paid.2010.01.024

[B55] Locke E. A. (2002). The dead end of postmodernism. *Am. Psychol.* 57:458 10.1037/0003-066X.57.6-7.458a 12094455

[B56] Luck S. J. Vogel E. K. (1997). The capacity of visual working memory for features and conjunctions. *Nature* 390 279–281. 10.1038/36846 9384378

[B57] Luck S. J. Vogel E. K. (2013). Visual working memory capacity: from psychophysics and neurobiology to individual differences. *Trends Cogn. Sci.* 17 391–400. 10.1016/j.tics.2013.06.006 23850263 PMC3729738

[B58] Ma W. J. Husain M. Bays P. M. (2014). Changing concepts of working memory. *Nature Neurosci.* 17 347–356. 10.1038/nn.3655 24569831 PMC4159388

[B59] Mandler G. (2007). *A History of Modern Experimental Psychology: From James and Wundt to Cognitive Science.* Cambridge, MA: MIT Press.

[B60] Martin J. (2003). Positivism, quantification and the phenomena of psychology. *Theory Psychol.* 13 33–38. 10.1177/0959354303013001760

[B61] Martin J. Sugarman J. (2009). Middle-ground theorizing, realism, and objectivity in psychology: a commentary on Held (2007). *Theory Psychol.* 19 115–122. 10.1177/0959354308101422

[B62] Matlin M. W. (2012). *Cognition*, 8th Edn Hoboken: Wiley.

[B63] McKenna M. Pereboom D. (2016). *Free Will: A Contemporary Introduction.* New York, NY: Routledge.

[B64] Mele A. R. (2007). “Decisions, intentions, urges, and free will: why libet has not shown what he says he has,” in *Causation and Explanation*, eds Campbell J. K. O’Rourke M. Silverstein H. S. (Cambridge, MA: MIT Press), 241–263.

[B65] Michell J. (2003). The quantitative imperative: positivism, naïve realism and the place of qualitative methods in psychology. *Theory Psychol.* 13 5–31. 10.1177/0959354303013001758

[B66] Nahmias E. (2010). “Scientific challenges to free will,” in *A Companion to the Philosophy of Action*, eds Sandis C. O’Connor T. (Malden: Wiley-Blackwell), 345–310. 10.1002/9781444323528.ch44

[B67] Nola R. Sankey H. (2014). *Theories of Scientific Method. An Introduction.* Stocksfield: Acumen.

[B68] O’Connor T. Franklin C. (2018). “Free will,” in *The Stanford Encyclopedia of Philosophy*, ed. Zalta E. N. Available online at: https://plato.stanford.edu/entries/freewill/

[B69] Olby R. C. Cantor G. N. Christie J. R. R. Hodge M. J. S. (eds). (1991). *Companion to the History of Modern Science.* London: Routledge.

[B70] Osterlind S. J. (2019). *The Error of Truth: How History and Mathematics Came Together to Form Our Character and Shape Our Worldview.* Oxford: OUP.

[B71] Parkin A. J. (2013). *Essential Cognitive Psychology* (classic edition). London: Psychology Press.

[B72] Paul L. A. Hall N. (2013). *Causation: A User’s Guide.* Oxford: OUP.

[B73] Pearl J. (2009). *Causality. Models, Reasoning, and Inference*, 2nd Edn Cambridge: CUP.

[B74] Petrie H. G. (1971). A dogma of operationalism in the social sciences. *Philos. Soc. Sci.* 1 145–160. 10.1177/004839317100100109

[B75] Pinker S. (2018). *Enlightenment Now. The Case for Reason, Science, Humanism, and Progress.* New York, NY: Viking.

[B76] Planck M. (1949). The meaning and limits of exact science. *Science* 110 319–327. 10.1126/science.110.2857.319 17770342

[B77] Radder H. Meynen G. (2013). Does the brain “initiate” freely willed processes? A philosophy of science critique of Libet-type experiments and their interpretation. *Theory Psychol.* 23 3–21. 10.1177/0959354312460926

[B78] Ramey H. L. Grubb S. (2009). Modernism, postmodernism and (evidence-based) practice. *Contemp. Fam. Ther.* 31 75–86. 10.1007/s10591-009-9086-6

[B79] Robinson M. M. Benjamin A. S. Irwin D. E. (2020). Is there a K in capacity? Assessing the structure of visual short-term memory. *Cogn. Psychol.* 121 101305. 10.1016/j.cogpsych.2020.101305 32531272

[B80] Ronzitti G. (ed.) (2011). *Vagueness: A Guide.* Dordrecht: Springer.

[B81] Roskies A. L. (2011). “Why Libet’s studies don’t pose a threat to free will,” in *Conscious Will and Responsibility*, eds Sinnott-Armstrong W. Nadel L. (Oxford: OUP), 11–22. 10.1093/acprof:oso/9780195381641.003.0003

[B82] Saigle V. Dubljević V. Racine E. (2018). The impact of a landmark neuroscience study on free will: a qualitative analysis of articles using Libet and colleagues’ methods. *AJOB Neurosci.* 9 29–41. 10.1080/21507740.2018.1425756

[B83] Schlosser M. E. (2014). The neuroscientific study of free will: a diagnosis of the controversy. *Synthese* 191 245–262. 10.1007/s11229-013-0312-2

[B84] Schurger A. (2017). “The neuropsychology of conscious volition,” in *The Blackwell Companion to Consciousness*, eds Schneider S. Velmans M. (Malden: Wiley Blackwell), 695–710. 10.1002/9781119132363.ch49

[B85] Schurgin M. W. (2018). Visual memory, the long and the short of it: a review of visual working memory and long-term memory. *Attention Percept. Psychophys.* 80 1035–1056. 10.3758/s13414-018-1522-y 29687357

[B86] Schurgin M. W. Brady T. F. (2019). When “capacity” changes with set size: ensemble representations support the detection of across-category changes in visual working memory. *J. Vis.* 19 1–13. 10.1167/19.5.3 31058989

[B87] Schurgin M. W. Cunningham C. A. Egeth H. E. Brady T. F. (2018). Visual long-term memory can replace active maintenance in visual working memory. *bioRxiv* [Preprint]. 10.1101/381848

[B88] Segerstråle U.C.O. (ed.) (2000). *Beyond the Science Wars: The Missing Discourse about Science and Society.* Albany: SUNY Press.

[B89] Seifert J. (2011). In defense of free will: a critique of Benjamin Libet. *Rev. Metaphys.* 65 377–407.

[B90] Shearer A. (2020). *The Story of Yoga: From Ancient India to the Modern West.* London: Hurst & Company.

[B91] Shepard J. (2018). How libet-style experiments may (or may not) challenge lay theories of free will. *AJOB Neurosci.* 9 45–47. 10.1080/21507740.2018.1425766

[B92] Sim S. (2011). *The Routledge Companion to Postmodernism*, 3rd Edn London: Routledge.

[B93] Sinacore A. L. Enns C. Z. (2005). “Diversity feminisms: postmodern, women-of-color, antiracist, lesbian, third-wave, and global perspectives,” in *Teaching and Social Justice: Integrating Multicultural and Feminist Theories in the Classroom*, eds Enns C. Z. Sinacore A. L. (Washington, DC: APA), 41–68. 10.1037/10929-003

[B94] Slavin R. E. (2002). Evidence-based education policies: transforming educational practice and research. *Educ. Res.* 31 15–21. 10.3102/0013189X031007015

[B95] Smedslund J. (2016). Why psychology cannot be an empirical science. *Integrative Psychol. Behav. Sci.* 50 185–195. 10.1007/s12124-015-9339-x 26712604

[B96] Sokal A. D. (1996). A physicist experiments with cultural studies. *Lingua Franca* 6 62–64.

[B97] Sternberg R. J. Sternberg K. (2011). *Cognitive Psychology*, 6th Edn Wadsworth: Cengage Learning.

[B98] Stroebe W. Strack F. (2014). The alleged crisis and the illusion of exact replication. *Perspect. Psychol. Sci.* 9 59–71. 10.1177/1745691613514450 26173241

[B99] Taleb N. N. (2007). *The Black Swan: The Impact of the Highly Improbable.* New York, NY: Random House.

[B100] Tarescavage A. M. (2020). Science Wars II: the insidious influence of postmodern ideology on clinical psychology (commentary on “Implications of ideological bias in social psychology on clinical practice”). *Clin. Psychol. Sci. Pract.* 27:e12319 10.1111/cpsp.12319

[B101] Teo T. (2018). *Outline of Theoretical Psychology.* London: Palgrave Macmillan.

[B102] Wax M. L. (1995). Method as madness science, hermeneutics, and art in psychoanalysis. *J. Am. Acad. Psychoanal.* 23 525–543. 10.1521/jaap.1.1995.23.4.525 8809719

[B103] Wegner D. M. (2017). *The Illusion of Conscious Will*, 2nd Edn Cambridge, MA: MIT Press.

[B104] Wilton R. Harley T. (2017). *Science and Psychology.* London: Routledge.

